# Static and Dynamic Impacts of Internet Use on Self-Rated Health among Adults in China: A Hybrid Model Analysis Based on National Panel Survey Data

**DOI:** 10.3390/ijerph20021003

**Published:** 2023-01-05

**Authors:** Mo Zhou, Isao Igarashi, Koichi Kawabuchi

**Affiliations:** Department of Health Care Economics, Graduate School of Medical and Dental Sciences, Tokyo Medical and Dental University, Tokyo 113-8549, Japan

**Keywords:** dynamic aspect, hybrid model, Internet use, panel data, self-rated health, static aspect

## Abstract

The widespread use of the Internet has a substantial impact on people’s livelihoods, including health-related factors. Whether this impact is beneficial or harmful to people’s health remains unclear. Some cross-sectional studies found static differences in the health status between Internet users and nonusers, whereas panel data studies found dynamic changes in an individuals’ health over time, making the issue, including its causality, controversial. Therefore, we aimed to clarify the association between the use of the Internet and people’s health from both static and dynamic aspects. Data were obtained for 46,460 adults from the China Family Panel Studies in 2014, 2016, and 2018. The analysis applied a logistic regression hybrid model with self-rated health as the dependent variable and Internet use as the main independent variable. In the hybrid model, time-varying independent variables were decomposed into between-individual (static) differences and within-individual (dynamic) changes over time. The results indicated that the between-individual coefficient of Internet use was significantly positive, but the within-individual coefficient was not, i.e., Internet users felt healthier than nonusers from the static aspect but starting to use the Internet did not increase the self-rated health from the dynamic aspect. These findings suggest that attention is needed in order to not confuse the static differences with dynamic change regarding the causality between Internet use and self-rated health.

## 1. Introduction

Recently, remarkable advances in Internet technology have been accompanied by rapid increases in the number of Internet users. In China, there were 1.03 billion Internet users in December 2021, an increase of 0.26 billion since December 2017. During these 4 years, the annual growth rate of Internet users was 7.5% and the penetration rate in the population rose from 55.8% to 73.0% [1]. Such widespread Internet use has a large impact on people’s livelihoods, including the health-related factors, because it enables an easier access to health information and health care, such as making doctor’s appointments, purchasing medication, and even receiving medical consultations online [2,3].

The Chinese government has attempted to improve the health level of the public through the Internet. A blueprint for improving the population’s health was released by the state council, the People’s Republic of China, in 2016, entitled “The Healthy China 2030” [4]. According to The Healthy China 2030, several policies have been proposed via the Internet to enhance the quality of medical services, information, and other aspects. Such policies aimed to (1) promote the integration of health, retirement, tourism, the Internet, and others; (2) develop Internet-based health services; (3) establish and promote a standard for “Internet + health care” services; (4) develop a smart information technology for medical use; and (5) comprehensively expand the application of large-scale health care data in the governance of industry, and for clinical and scientific research, public health, and education.

In addition, regular Internet users are more likely than non-regular Internet users to live a healthier lifestyle [5], and frequent Internet users have shown better self-rated health than less frequent users [6]. However, Internet use is also associated with a number of side effects. Some cross-sectional studies have found that heavy Internet users engage in fewer health-promoting behaviors and more risky behaviors than do light Internet users [7] and have poorer self-rated mental health [8]. Further, users addicted to the Internet have shown worse physical, mental, and social health [9]. Meanwhile, a study found that the increasing frequency of using the Internet had no significant effect on one’s depression levels using a panel fixed-effects model (an estimator of the panel fixed-effects model is also known as a within-estimator) [10]. Therefore, the association between the use of the Internet and the users’ health remains controversial. There is clearly a need for further research regarding the relationship between the use of the Internet and an individuals’ health status.

It is well known that cross-sectional analyses can reveal differences between survey subjects but cannot detect causality. By contrast, panel data analysis, especially using a fixed-effects model, can handle changes in variables within survey subjects over time, which approaches a causal relationship more closely than cross-sectional analyses. However, fixed-effects models fully exclude the components of differences between survey subjects from the analysis, and this prevents the association of variation across subjects since the outcomes are undetectable [11]. Therefore, the purpose of this study was to clarify the association between Internet usage and health among the public in China from two aspects: “static” differences and “dynamic” changes over time. In this study, the static aspect identifies the effect of the independent variables on the dependent variable between individuals at a point in time. The dynamic aspect identifies the effect of the independent variables on the dependent variable within individuals over time. As a consequence, the results of the static aspect are similar to those of the cross-sectional data analysis, which indicates the differences between individuals. The results of the dynamic aspect are equivalent to those of the panel fixed-effects analysis, which indicate the changes within individuals over time. Based on previous studies [5,6,7,8,9,10], there is still ambiguity about how the use of the Internet affects one’s health status. Thus, in this study, we intend to examine whether there are differences in the self-rated health between Internet users and nonusers in the static aspect. Additionally, while “The Healthy China 2030” policy has proposed using the Internet to improve the health status of the population, it should be investigated whether the use of the Internet improves the population’s health over time from the dynamic aspect.

Concretely, we hypothesized the following: (1) Internet users are in better health than nonusers (from the aspect of static variation), and (2) starting to use the Internet (changing from a non-Internet user to an Internet user) improves an individuals’ health status (from the aspect of dynamic change). The findings of these research projects are expected to contribute to the development of the Chinese public health policy.

## 2. Materials and Methods

### 2.1. Data and Sample

We obtained survey data from the China Family Panel Studies (CFPS). These have been conducted biennially by the Institute of Social Science Survey at Peking University, since 2010. The CFPS are national longitudinal social surveys conducted to investigate the recent changes in the Chinese society, economy, population, education, and health. The data cover 25 provinces, municipalities, and autonomous regions in China (excluding Hong Kong, Macao, Taiwan, Xinjiang, Tibet, Qinghai, Inner Mongolia, Ningxia, and Hainan), which contain 95% of China’s population; therefore, it is nationally representative in substance [12]. In the CFPS, the target sample was 16,000 households and included all individuals living in those households, consisting of both family and nonfamily members, such as domestic helpers. The CFPS is a panel survey that, in principle, tracks family members in the subsequent survey. However, some individuals drop out because of reasons such as death or moving out of the community, while newborn and adopted children are added. The CFPS data are available to academic researchers and public policymakers [13]. We used the CFPS adult dataset for three waves in 2014, 2016, and 2018, in which the subjects were aged 16 years and over. In each of the three waves, “self-rated health”, one of the questionnaire’s items of concern, was coded in the same way. The numbers of adult respondents in 2014, 2016, and 2018 were 37,147, 36,892, and 37,354, respectively [14,15,16]. The total number of adult respondents throughout the three waves was 46,896 after verifying identical persons.

Before the CFPS interviews, the interviewers provided explanations about the survey, especially in regard to its confidentiality, to every participant [17], and those who consented to answer participated in the survey [13].

### 2.2. Dependent Variable

As the outcome indicator, we used self-rated health, which has frequently been used as a health measure [18,19,20,21]. The question item for self-rated health was “How would you rate your health status?”, and the answer choices were “Excellent”, “Very good”, “Good”, “Fair”, and “Poor” [22]. This five-point scale was transformed into a dummy variable, which was assigned a score of 1 for responses of “Excellent”, “Very good”, or “Good”, and 0 otherwise, for entry into binary logistic regression analysis in line with a previous study [23].

### 2.3. Independent Variables

As the key issue of this study was the influence of Internet usage on self-rated health, we set the time-varying use of the Internet as the main independent variable (use = 1, do not use = 0). For the other independent variables, we selected 13 kinds of time-varying and two kinds of time-invariant variables from the questionnaire items. The 13 kinds of time-varying variables consisted of five dummy variables, two ordinal variables, five continuous variables, and one categorical variable. The five dummy variables were marital status (married/have a spouse/cohabiting = 1, otherwise = 0; “otherwise = 0” is omitted hereafter), smoking (smoked cigarettes in the past month = 1), alcohol drinking (drank alcohol at least three times a week in the past month = 1), public health insurance (enrolled = 1), and residential area (urban = 1, rural = 0). The two ordinal variables were self-rated relative to income and self-rated social status, both of which were on a five-point scale from 1 (very low) to 5 (very high). We divided each ordinal variable into five dummy variables by points. The five continuous variables were daily sleeping hours, the frequency of physical exercise in the past week, personal income (CNY; 1 CNY ≈ 0.14 USD), and height and weight. We created the square term of daily sleeping hours and transformed the participants’ income logarithmically. From the participants’ height and weight, we calculated the body mass index (BMI) and created two dummy variables: “overweight” (more than 25.0 kg/m^2^ = 1) and “underweight” (less than 18.5 kg/m^2^ = 1). We changed the one categorical variable, educational attainment, into a continuous variable: “years of education”. For example, we converted “high school graduates” into 12 years of education. The two kinds of time-invariant variables were the respondents’ age in 2016 (equivalent to birth cohort) and his/her gender dummy (man = 1). We also created the square term of the respondents’ ages. In addition to the abovementioned variables, we also created two survey wave dummies: wave in 2016 and wave in 2018.

Moreover, time-varying independent variables, including Internet usage, were decomposed into two components: between-individual differences and within-individual changes. In the panel dataset, the identical individuals were repeatedly observed over three waves, in essence. First, we calculated the means of each time-varying variable over time for every individual, which represented the between-individual differences (i.e., static differences) in the variable-like values of the cross-sectional data. Second, we subtracted the individual-specific means from the observed values of each variable for every individual, which represented the within-individual changes (i.e., dynamic changes) in each variable over time [11].

### 2.4. Statistical Analyses

Among the 46,896 adult respondents in the three waves, 46,886 provided answers on self-rated health at least once, 35,552 two or three times, and 22,091 three times. Meanwhile, many missing values were seen throughout the dataset, so we performed multiple imputation (MI) to fill in the missing values. The MI procedure fills in each missing value with a plausible value estimate based on all of the non-missing values of all of the variables in the dataset [24,25]. We repeated the random imputation process 100 times, and then 100 imputed datasets were generated. After 100 imputation processes, all of the variable transformations mentioned above were made, such as binarizing ordinal variables, creating square terms, performing log-transformations, preparing new dummy variables, and decomposing time-varying variables.

Subsequently, using the 100 imputed datasets, we conducted two logistic regression models—a null model and a full model—with binarized self-rated health as the dependent variable. The null model had no independent variables except the constant terms. In the full model, the independent variables were the between- and within-individual variables derived from the use of the Internet and 13 other kinds of time-varying variables, two kinds of time-invariant variables, and two survey wave dummies. A panel data analysis model with both between- and within-individual independent variables is called a hybrid model [11]. Furthermore, the square terms of daily sleeping hours and age were entered into the full model together with the linear terms. Concurrently, the log-transformed income, overweight and underweight dummies, and dummy variables divided from the ordinal scales were entered instead of the respondents’ income, their height and weight, and ordinal scales of self-rated relative income and social status, in that order.

In the context of MI, a regression analysis is conducted separately on each imputed dataset, and the 100 regression results are combined into a single result [24,25]. All statistical procedures were performed using Stata release 16.1 (StataCorp, College Station, TX, USA).

## 3. Results

### 3.1. Descriptive Statistics

Regarding the descriptive statistics (Table 1), among 37,147, 36,892, and 37,354 respondents in 2014, 2016, and 2018, respectively, over 30,000 valid responses were received, covering most of the questionnaire items. However, fewer than 30,000 respondents validly answered the item for their income in 2016 and in 2018, educational attainment in 2018, self-rated relative income in 2014 and 2018, public health insurance in 2018, and both height and weight (relevant to their BMI) in 2018. Regarding self-rated health, the percentages of those who answered “Excellent”, “Very good”, or “Good” averaged about 70% over the three waves. The percentage of Internet usage showed an increasing trend, from 29.9% in 2014 to 53.1% in 2018.

The mean age of the respondents was around 46 years over the three waves. From 2014 to 2018, education and the frequency of physical exercise in the past week increased from 7.5 to 8.2 years and from 1.8 to 2.6 times, respectively. The mean percentage of those who resided in an urban area was about 48% until 2016 and increased to 51% in 2018. The respondents’ daily sleeping time was approximately 7.8 h throughout the three waves. Those who were married, had a spouse, or cohabited accounted for more than 70% of the respondents. Those who had smoked in the past month, had drunk more than three times a week in the past month, and who were enrolled in public health insurance accounted for more than 70%, about 15%, and about 91% of respondents in each wave, respectively. The prevalence of those who were overweight increased from 22.5% in 2014 to 26.5% in 2018, while those of the respondents who were a normal weight and underweight decreased from 68.0% to 64.9% and from 9.5% to 8.7% during the same period, respectively. The mean income was volatile: it was CNY 9022.7 in 2014, CNY 21,768.4 in 2016, and CNY 18,803.3 in 2018. Regarding the self-rated relative income, the percentage of those who answered “Very high” or “High” averaged around 10% until 2016 and increased to 22.9% in 2018. Conversely, those who answered “Low” or “Very low” averaged between 43% and 51% until 2016, and then decreased to 30.1% in 2018. Regarding the self-rated social status, no clear trend was observed, as the percentage of those who answered “Very high” or “High” varied from 20.2% to 29.7%, while the percentage of those who answered “Low” or “Very low” varied from 24.1% to 34.2%.

### 3.2. Regression Results

The results of the null model (Table 2) revealed a ρ value of 0.594, which means that the within-individual components (dynamic aspect) determined 40.6% (=1 − 0.594) of the total variance of the dependent variable (self-rated health), and the between-individual components (static aspect) 59.4%.

The results of the hybrid model with MI (Table 3) showed that the between-individual coefficient was 0.342 and significant regarding the main independent variable, Internet usage, while the within-individual coefficient was positive and not significant. Regarding smoking and years of education, the between-individual coefficients were significantly positive and the within-individual coefficients were insignificantly positive. Besides, the overweight dummy had a significantly negative between-individual coefficient and a non-significantly negative within-individual coefficient. Regarding the drinking, sleeping hours, physical exercise, log-transformed income, the four dummies of the self-rated income level, and the four dummies of the self-rated social status, both the between- and within-individual coefficients were significantly positive, but only the within-individual coefficient for drinking was significant at the 10% level. In addition, the largest coefficient value of the self-rated income level was very high, followed by high, medium, and low, in that order, regarding both the between- and within-individual components. The coefficient values of the self-rated social status also aligned in the same order in both aspects of the between- and within-individual components. The underweight dummy and the square of sleeping hours were significantly negative regarding both the between- and within-individual coefficients. Based on the coefficients of the linear and square terms, the within- and between-individual sleeping hours were positively correlated with the dependent variable for less than 9.2 and 8.5 h, respectively, and negatively correlated for more than 9.2 and 8.5 h, respectively. Public health insurance had a non-significantly negative between-individual coefficient and a significantly positive within-individual coefficient at the 10% level. Regarding variables with no distinction between the between- and within-individual components, the male gender and the square of age had significantly positive coefficients, and age had significantly negative coefficients. The respondents’ age was negatively correlated with the dependent variable at younger than 90.3 years and positively at older than 90.3 years.

### 3.3. Sensitivity Analysis

Regarding the sensitivity analysis, we conducted another full model without MI (Table 4). Comparing the results of the two full models with and without MI, the number of observations increased from 53,113 to 139,376, and the number of individuals rose from 34,073 to 46,460 by MI. No gaps between the coefficient estimators of both models were observed. All of the standard errors were reduced after MI, probably because of the increased number of observations. Consequently, four insignificant within-individual coefficients before MI—public health insurance, a low self-rated income level, the frequency of physical exercise, and log-transformed income—became significant after MI. Additionally, the within-individual coefficient of a very high self-rated social status, which had been significant at the 5% level, became significant at the 1% level. However, only the within-individual coefficient of the overweight dummy, which had been significant before MI, became non-significant after MI. As a whole, the results of the full model with MI were similar to those of the model without MI.

## 4. Discussion

In this study, panel data analyses conducted with CFPS datasets in 2014, 2016, and 2018 with a null model revealed that self-rated health was determined by changes in individual attributes over time (the dynamic aspect), and variation in individual attributes (the static aspect), at a ratio of approximately 4 to 6. Both aspects of the attributes were effective in measurable proportions, so we employed a hybrid model in which time-varying determinants were decomposed into within- and between-individual components. The between-individual coefficient of Internet use was significant, at 0.342, and the odds ratio (OR) was 1.41 (the OR is an exponential of the coefficient). However, the within-individual coefficient of Internet use was positive and not significant, which means that Internet users are statically 1.41 times more confident of their self-rated health than are nonusers, while starting to use the Internet is not dynamically effective in improving the self-rated health. Consequently, hypothesis (1) was supported, but not hypothesis (2).

Among the previous literature about the association of the use of the Internet with one’s health status, to our knowledge, self-rated health is often seen as the indicator of an individuals’ health status along with their mental health (including depression). Unlike various biological indicators, self-rated health is an integrated variable and a holistic assessment of an individual’s health status [18,19,20]. Therefore, we chose self-rated health as the health indicator in this study.

Previous cross-sectional studies with the CFPS dataset in 2018 showed that Internet users indicated a better self-rated health than did nonusers among adults [23,26]. Additionally, a previous study that conducted panel analyses, in which lagged dependent and independent variables (i.e., self-rated health and the Internet usage of the preceding wave, respectively) were entered as the independent variables, using the CFPS data from 2014 to 2018, and found that the use of the Internet has a significantly positive association with self-rated health among adults [27]. Although the kind of panel analysis model of the study was not clearly described, it seems to be a random-effects model. In the present and the previous studies, the origin of the data was the same (CFPS dataset). However, unlike previous studies, we performed both the within- and between-estimation simultaneously. The between-estimation is similar to a cross-sectional estimation using the individual-specific means of the variables measured longitudinally instead of cross-sectionally, at a point in time. In the present study, the results deduced from the between-individual coefficient of Internet use were the same as those of the two previous cross-sectional studies. On the other hand, the results obtained with the within-individual coefficient of Internet use were different from that of the previous panel study. However, the results of the random-effects model which we preliminarily conducted showed that the coefficient of Internet use was significantly positive and consistent with that of the previous panel study. In theory, the coefficients of a fixed-effects (within-estimation) model are always unbiased, and the coefficients of a random-effects model are intermediate between the between-estimator and within-estimator [28]. That is, the results of a random-effects model are not always unbiased. Therefore, we believe the results of the within-individual coefficients of the present study may possibly be more reliable than the results of the previous panel study. Further study is required to make the point clear.

For reference, previous studies with the dependent variable except self-rated health have reported that after 3 years of Internet usage, individuals rate themselves as having better physical/cognitive health and social well-being and attending more health screenings compared with nonusers [29], whereas those who had used the Internet heavily at the age of 18 years had worse mental health at the ages of 21–22 years [30]. In addition, a study using panel data in a random-effects model reported that the use of the Internet was associated with a lower risk of a depressed state [31]. Moreover, a study that conducted panel fixed-effects analyses with datasets from the two waves of the CFPS in 2016 and 2018 found that changing from a non-Internet user to an Internet user reduced depression in older adults aged 60 years and over [32].

As a whole, a major finding of this study is that a causal relationship could not be inferred from starting to use the Internet to feeling healthier regardless of the different self-rated health status between Internet users and nonusers. In other words, it leaves open the possibility that present Internet users may have felt healthy before starting to use the Internet, and that those who have been feeling unhealthy may be unwilling to start using the Internet. These findings were achieved because of the strength of the hybrid model, which simultaneously analyzed two aspects, i.e., the dynamic change over time and static divergence, and approached exact causal inferences closer than possible with a conventional analytical design.

In 2016, the Chinese government announced The Healthy China 2030, in Chapter 24 of which, “internet + health care” services were proposed to cover people’s whole life cycle health management [4]. Of course, it is expected that the measure is beneficial to present Internet users. However, based on the results of this study, it is questionable whether the measure will be sufficiently effective in improving the present non-Internet users’ health status even when they will start using the Internet. In this sense, the measures depending on the recent rapid increase in Internet users may not work enough to promote the overall health status of the people. Some alternative measures may be required to reduce the health disparities between Internet users and nonusers.

Contrary to our expectations, the between-individual coefficients of both smoking and drinking were significantly positive, which indicates that those who had smoked in the past month and those who had drunk over three times a week in the past month rated their health status as higher than those who had not. In addition, the within-individual coefficient of drinking was positively significant at the 10% level, which implies that self-rated health increases with the increasing frequency of drinking. This may be explained by unhealthy behavior leading to overrated self-reported health. Indeed, heavy smokers have reported excellent health as a result of an overconfidence in their own health [33]. Moreover, heavy drinkers aged 45 years and older have reported a better health status than those who only drink occasionally [34].

Meanwhile, we found from the results of the between-individual coefficients that those whose daily sleeping hours were around 8.5 h had better self-rated health than those who had shorter or longer daily sleeping hours. We also found from the results of the within-individual coefficients that, if someone slept fewer than 9.2 h, prolonging their sleeping time made him/her feel healthier, and if they slept for more than 9.2 h, shortening their sleeping time made him/her feel healthier. Previous studies have reported that short or insufficient sleep is associated with worse self-rated health [35,36]. The results of the present study indicated the effects of sleeping for too long and for too little using the square term. Indeed, a sleep duration of 7–8 h was associated with the lowest risk of chronic diseases such as obesity, diabetes, hypertension, and cardiovascular disease [37].

In addition, both being overweight and underweight were associated with a poor self-rated health, and recovery from being underweight improved one’s self-rated health. Moreover, those who engaged in frequent physical exercise reported a better self-rated health than those who did not, and increasing the frequency of physical exercise improved their self-rated health. Furthermore, the non-significant between-individual coefficient of public health insurance indicated that both enrollees and non-enrollees in public health insurance were homogeneous in terms of their self-rated health. However, the within-individual coefficient suggested that enrolling in public health insurance significantly improved one’s self-rated health at the 10% level.

Regarding socioeconomic status, we found that one’s income and two indices of subjective socioeconomic status (the self-rated income level and self-rated social status) were positively associated with self-rated health from both the between- and within-individual (static and dynamic) aspects. That is, from the static aspect, those who had a high income, perceived themselves as having a high income, and those who perceived themselves as having a high social status felt healthier than those who did not. A previous cross-sectional study reported that both the income and subjective identification of socioeconomic status were positively associated with self-rated health in China [38]. From the dynamic aspect, a real increase in one’s income, the perception of receiving a higher income, and the perception of increasing one’s social status improved the self-rated health of the participants.

Along with income, education is an index of real socioeconomic status. In this study, the statistically positive between-individual coefficient of years of education meant that highly educated persons had a better self-rated health than the less educated. This finding is consistent with those from previous cross-sectional studies [26,39]. However, the non-significant within-individual coefficient does not support the hypothesis that obtaining a higher education is related to self-rated health.

This study has several limitations. First, the results do not necessarily reflect the viewpoints of healthcare providers or professionals because we used national social survey data and, consequently, focused on the use of the Internet among the general public. Second, the purpose or frequency of the use of the Internet was not considered. For example, searching for health information online may have positive effects on one’s self-rated health, while lengthy hours of Internet usage for entertainment may have negative effects. However, in the present study, we did not differentiate the kinds of purposes or the ranks of frequency, so the positive and negative effects may have been mixed and canceled each other out through the statistical analysis. Consequently, the within-individual coefficient of Internet use may seem to be non-significant. Further research is required to address this issue. Third, the association between the use of the Internet and self-rated health may have been affected by the attributes of the respondents, such as their age, gender, and socioeconomic status. For example, young people may tend to be enthusiastic about entertainment, whereas older adults may search for health-care information more often. However, differences in the associations by attributes may have been missed because regression analyses were not performed separately by attributes. Fourth, the precision of the data for the dependent variable, self-rated health, had to be reduced from a five-point scale to a binary item because a panel ordinal logistic regression analysis of multiple imputed data is not possible in Stata.

## 5. Conclusions

In the present study, the association between the use of the Internet and self-rated health was analyzed using a hybrid model with national social survey data from 2014 to 2018 among adults in China. The between-individual coefficient (the static aspect) of Internet use showed that Internet users were statistically 1.41 times more confident of their self-rated health. However, the within-individual coefficient (the dynamic aspect) of Internet use did not indicate the dynamic effectiveness of starting to use the Internet for improving one’s self-rated health. In other words, the increase in Internet users may not improve the individuals’ health status. These results provide a concrete example of how cross-sectional analysis is deficient in proving causality between two variables. In addition, our findings suggest that policy makers should pay close attention to not confuse the static variation with dynamic change when considering the causal relationship between the use of the Internet and self-rated health. If policymakers attempt a public health promotion by increasing the number of Internet users, it may be hard to achieve the desired results. Rather, since Internet users have a higher health status than nonusers, it is necessary to discuss how to reduce the health disparities between Internet users and nonusers.

## Figures and Tables

**Table 1:** Descriptive statistics before multiple imputation.

Total Number of Respondents Over the Three Waves: 46,896									
Wave		2014			2016			2018	
Number of Respondents	37,147			36,892			37,354		
Continuous Variables	n	Mean	SD	n	Mean	SD	n	Mean	SD
Age (years)	36,861	45.3	17.4	36,553	46.0	17.7	30,194	46.4	17.1
Daily sleeping hours	32,873	7.9	1.5	32,912	7.8	1.5	30,319	7.8	1.5
Income (CNY)	36,694	9022.7	18,884.6	9715	21,768.4	39,472.4	24,402	18,803.3	33,037.6
Years of education	37,082	7.5	4.8	34,421	7.7	4.8	26,936	8.2	5.0
Physical exercise ^(1)^	33,452	1.8	2.9	33,222	2.1	3.0	30,460	2.6	3.1
Categorical variables	Frequency		%	Frequency		%	Frequency		%
Self-rated health	37,123		100.0	36,882		100.0	30,514		100.0
Excellent	5484		14.8	4963		13.5	4211		13.8
Very good	7850		21.1	6834		18.5	4826		15.8
Good	12,624		34.0	12,786		34.7	12,598		41.3
Fair	5378		14.5	6434		17.4	3961		13.0
Poor	5787		15.6	5865		15.9	4918		16.1
Self-rated relative income	28,952		100.0	30,574		100.0	28,253		100.0
Very high	975		3.4	1114		3.6	2712		9.6
High	2197		7.6	1800		5.9	3772		13.4
Medium	13,208		45.6	12,325		40.3	13,274		47.0
Low	6996		24.2	8341		27.3	5250		18.6
Very low	5576		19.3	6994		22.9	3245		11.5
Self-rated social status	31,450		100.0	33,128		100.0	30,062		100.0
Very high	2092		6.7	2495		7.5	3718		12.4
High	5053		16.1	4205		12.7	5206		17.3
Medium	15,872		50.5	15,091		45.6	13,893		46.2
Low	5248		16.7	6631		20.0	4595		15.3
Very low	3185		10.1	4706		14.2	2650		8.8
Internet use	31,591		100.0	33,243		100.0	30,516		100.0
Yes	9443		29.9	14,553		43.8	16,212		53.1
No	22,148		70.1	18,690		56.2	14,304		46.9
Gender	37,110		100.0	36,832		100.0	30,475		100.0
Male	18,553		50.0	18,377		49.9	15,146		49.7
Female	18,557		50.0	18,455		50.1	15,329		50.3
Marital status	37,131		100.0	36,886		100.0	30,169		100.0
Married, etc. ^(2)^	28,428		76.6	28,164		76.4	23,764		78.8
Otherwise	8703		23.4	8722		23.7	6405		21.2
Smoking last month	31,590		100.0	33,242		100.0	30,515		100.0
Yes	8986		28.4	9252		27.8	8733		28.6
No	22,604		71.6	23,990		72.2	21,782		71.4
Alcohol drinking	31,589		100.0	33,243		100.0	30,514		100.0
More than three times ^(3)^	4818		15.3	4774		14.4	4539		14.9
Otherwise	26,771		84.7	28,469		85.6	25,975		85.1
Public health insurance	35,899		100.0	36,719		100.0	29,970		100.0
Enrolled	32,681		91.0	33,411		91.0	27,511		91.8
Not enrolled	3218		9.0	3308		9.0	2459		8.2
Residential area	34,226		100.0	36,591		100.0	30,009		100.0
Urban	16,349		47.8	17,530		47.9	15,300		51.0
Rural	17,877		52.2	19,061		52.1	14,709		49.0
BMI	31,735		100.0	30,321		100.0	29,389		100.0
Overweight	7147		22.5	7390		24.4	7775		26.5
Normal weight	21,568		68.0	20,090		66.3	19,061		64.9
Underweight	3020		9.5	2841		9.4	2553		8.7

Notes: many missing values were seen in the data, so multiple imputation was performed. N: the number of respondents who answered the questionnaire item. SD: standard deviation. (1) Physical exercise: frequency of physical exercise in the past week. (2) Married, etc.: married, have a spouse, or cohabitate. (3) More than three times: drinking more than three times in the past week.

**Table 2:** Results of the null model.

Model: Logistic regression model with multiple imputations Dependent variable: self-rated health (binary) Number of imputations: 100 Number of observations: 140,398 Number of individuals: 46,881			
	Coefficient	Standard Error	*p*-Value
Constant	1.437 **	0.016	0.000
σ_u_	2.193	0.022	
*ρ*	0.594	0.005	

Notes: the *ρ* values indicate that the between-individual component (static aspect) determined 59.4% of the total variance of the dependent variable, and the within-individual component (dynamic aspect) 40.6%. ** *p* < 0.01. *ρ* = σ_u_/(σ_e_ + σ_u_). σ_u_: variance of the between-individual component. σ_e_: variance of the within-individual component.

**Table 3:** Results of the hybrid model.

Model: logistic regression hybrid model with multiple imputation Dependent variable: self-rated health (binary) Number of imputations: 100 Number of observations: 139,376 Number of individuals: 46,460								
	Between-Individual				Within-Individual			
Individual Time-Varying Variables	Coefficient		Standard Error	*p*-Value	Coefficient		Standard Error	*p*-Value
Internet use								
Yes	0.342	**	0.047	0.000	0.039		0.034	0.249
No (reference)	—		—	—	—		—	—
Marital status								
Married, etc. ^(1)^	−0.059		0.045	0.186	0.005		0.072	0.950
Others (reference)	—		—	—	—		—	—
Smoking last month								
Yes	0.167	**	0.038	0.000	0.058		0.058	0.314
No (reference)	—		—	—	—		—	—
Alcohol drinking last month								
More than three times a week	0.471	**	0.049	0.000	0.078	†	0.046	0.089
Others (reference)	—		—	—	—		—	—
Public health insurance								
Enrolled	−0.098		0.077	0.208	0.076	†	0.043	0.080
Not enrolled (reference)	—		—	—	—		—	—
Residential area								
Urban	0.016		0.028	0.576	−0.060		0.055	0.268
Rural (reference)	—		—	—	—		—	—
Self-rated income level								
Very high	1.402	**	0.107	0.000	0.494	**	0.059	0.000
High	1.170	**	0.104	0.000	0.423	**	0.059	0.000
Medium	1.035	**	0.060	0.000	0.276	**	0.035	0.000
Low	0.460	**	0.067	0.000	0.112	**	0.042	0.008
Very low (reference)	—		—	—	—		—	—
Self-rated social status								
Very high	1.042	**	0.096	0.000	0.317	**	0.050	0.000
High	0.755	**	0.083	0.000	0.287	**	0.057	0.000
Medium	0.445	**	0.071	0.000	0.221	**	0.038	0.000
Low	0.261	**	0.079	0.001	0.197	**	0.038	0.000
Very low (reference)	—		—	—	—		—	—
Sleeping hours	1.212	**	0.082	0.000	0.324	**	0.049	0.000
Sleeping hours^2^	−0.072	**	0.005	0.000	−0.018	**	0.003	0.000
Frequency of physical exercise	0.060	**	0.006	0.000	0.012	**	0.004	0.002
Ln income	0.021	**	0.004	0.000	0.016	**	0.003	0.000
Years of education	0.045	**	0.003	0.000	0.015		0.011	0.176
BMI								
Overweight	−0.174	**	0.033	0.000	−0.038		0.035	0.280
Normal weight (reference)	—		—	—	—		—	—
Underweight	−0.697	**	0.054	0.000	−0.141	*	0.061	0.022
Other Variables	Coefficient		Standard Error	*p*-Value				
Age	−0.122	**	0.005	0.000				
Age^2^	0.001	**	0.000	0.000				
Gender								
Male	0.164	**	0.031	0.000				
Female (reference)	—		—	—				
Wave in 2016	−0.339	**	0.020	0.000				
Wave in 2018	−0.214	**	0.023	0.000				
Constant	−1.037	**	0.342	0.002				
σ_u_	1.650		0.018					
*ρ*	0.453		0.005					

Notes: the between-individual coefficient of Internet use was significantly positive, so Internet users indicated better self-rated health than did nonusers. The within-individual coefficient of Internet use was not significant, so the change from nonuser to user did not improve self-rated health. (1) Married, etc.: married, have a spouse, or cohabitate. ** *p* < 0.01; * *p* < 0.05; † *p* < 0.1. *ρ* = σ_u_/(σ_e_ + σ_u_). σ_u_: variance of the between-individual component. σ_e_: variance of the within-individual component. —: reference.

**Table 4:** Results of the hybrid model without multiple imputation.

Model: logistic regression hybrid model without multiple imputation Dependent variable: self-rated health (binary) Number of observations: 53,113 Number of individuals: 34,073								
	Between-Individual				Within-Individual			
Individual Time-Varying Variables	Coefficient		Standard Error	*p*-Value	Coefficient		Standard Error	*p*-Value
Internet use								
Yes	0.250	**	0.058	0.000	0.048		0.060	0.428
No (reference)	—		—	—	—		—	—
Marital status								
Married, etc. ^(1)^	−0.071		0.055	0.194	−0.105		0.119	0.375
Others (reference)	—		—	—	—		—	—
Smoking last month								
Yes	0.195	**	0.049	0.000	−0.034		0.092	0.711
No (reference)	—		—	—	—		—	—
Alcohol drinking last month								
More than three times a week	0.400	**	0.058	0.000	0.135		0.072	0.060
Others (reference)	—		—	—	—		—	—
Public health insurance								
Enrolled	−0.043		0.082	0.600	0.070		0.078	0.369
Not enrolled (reference)	—		—	—	—		—	—
Residential area								
Urban	0.018		0.038	0.637	−0.112		0.098	0.252
Rural (reference)	—		—	—	—		—	—
Self-rated income level								
Very high	1.333	**	0.129	0.000	0.589	**	0.105	0.000
High	1.009	**	0.103	0.000	0.470	**	0.090	0.000
Medium	0.980	**	0.069	0.000	0.255	**	0.063	0.000
Low	0.424	**	0.074	0.000	0.074		0.062	0.233
Very low (reference)	—		—	—	—		—	—
Self-rated social status								
Very high	0.848	**	0.114	0.000	0.241	*	0.100	0.016
High	0.601	**	0.096	0.000	0.284	**	0.088	0.001
Medium	0.439	**	0.080	0.000	0.267	**	0.075	0.000
Low	0.270	**	0.090	0.003	0.206	**	0.078	0.008
Very low (reference)	—		—	—	—		—	—
Sleeping hours	1.001	**	0.086	0.000	0.241	**	0.086	0.005
Sleeping hours^2^	−0.059	**	0.005	0.000	−0.013	*	0.005	0.019
Frequency of physical exercise	0.049	**	0.007	0.000	0.007		0.007	0.356
Ln income	0.017	**	0.005	0.000	0.007		0.005	0.191
Years of education	0.058	**	0.005	0.000	0.011		0.038	0.775
BMI								
Overweight	−0.271	**	0.043	0.000	−0.205	**	0.069	0.003
Normal weight (reference)	—		—	—	—		—	—
Underweight	−0.825	**	0.072	0.000	−0.231	*	0.097	0.018
Other Variables	Coefficient		Standard Error	*p*-Value				
Age	−0.155	**	0.008	0.000				
Age^2^	0.001	**	0.000	0.000				
Gender								
Male	0.223	**	0.045	0.000				
Female (reference)	—		—	—				
Wave in 2016	−0.303	**	0.046	0.000				
Wave in 2018	−0.250	**	0.033	0.000				
Constant	0.725	†	0.387	0.061				
σ_u_	1.702		0.039					
*ρ*	0.468		0.011					

Notes: the results were similar between the two hybrid models with and without multiple imputation as a whole. (1) Married, etc.: married, have a spouse, or cohabitate. ** *p* < 0.01; * *p* < 0.05; † *p* < 0.1. *ρ* = σ_u_/(σ_e_ + σ_u_). σ_u_: variance of the between-individual component. σ_e_: variance of the within-individual component. —: reference.

## Data Availability

The CFPS data are available at https://www.isss.pku.edu.cn/cfps/ (accessed on 1 December 2022).
